# Lack of effect of apolipoprotein C3 polymorphisms on indices of liver steatosis, lipid profile and insulin resistance in obese Southern Europeans

**DOI:** 10.1186/1476-511X-10-93

**Published:** 2011-06-10

**Authors:** Federica Sentinelli, Stefano Romeo, Cristina Maglio, Michela Incani, Maria A Burza, Francesca Scano, Federica Coccia, Efisio Cossu, Frida Leonetti, Marco G Baroni

**Affiliations:** 1 Endocrinology and Diabetes, Department of Medical Sciences, University of Cagliari, Italy; 2 University of Cambridge Metabolic Research Laboratories, Institute of Metabolic Science, Addenbrooke's Hospital, Cambridge, UK; 3 Department of Clinical Sciences, University of Rome La Sapienza, Rome, Italy; 4 University of Gothenburg, Department of Molecular and Clinical Medicine, Sahlgrenska Center for Cardiovascolar and Metabolic Research, Sweden

**Keywords:** hepatic transaminases, NAFLD, BMI, obesity, tryglicerides, HDL, atherogenic dyslipidemia

## Abstract

**Background:**

Apolipoprotein C3 (APOC3) is a component of triglyceride-rich lipoproteins, and APOC3 rs2854116 and rs2854117 polymorphisms have been associated with non-alcoholic fatty liver disease, hypertriglyceridaemia, and insulin-resistance.

**Objective:**

To determine if the APOC3 variants alter the susceptibility of obese subjects to develop liver damage, hypertrigliceridaemia, and insulin-resistance.

**Methods:**

The study was carried out on 585 unrelated obese Italians (median body mass index BMI = 41 kg/m^2^) who were genotyped for the rs2854116 and rs2854117 variants. All participants underwent oral glucose tolerance tests (OGTT), with measurement of glucose, insulin, lipid parameters. Indices of insulin-resistance (HOMA and ISI) were calculated. Alanine transaminase (ALT) and aspartate transaminase (AST) were used as markers of liver injury.

**Results:**

The study subjects were divided into two groups: those homozygous for the wild-type alleles at both SNPs (-482C and -455T alleles) and those who were carriers of at least one variant allele or both (-482T, -455C or both). Also each SNP was analysed independently. No significant differences were found in ALT and AST levels and in the lipid profile between the two groups. Insulin concentrations, glucose tolerance and insulin sensitivity were similar in the two groups.

**Conclusion:**

We did not identify any significant association between APOC3 polymorphisms and fatty liver disease, lipids, and insulin-resistance in obese subjects, thus not confirming the suggested role of these APOC3 gene sequence variants.

## Background

Non-alcoholic fatty liver disease (NAFLD) is a multifactorial disorder arising from the interplay between genetic susceptibility and environmental influences. A large body of evidence shows that NAFLD is highly related to obesity and its metabolic consequences such as insulin resistance and dyslipidaemia [1]. In addition to altering metabolic risk, hepatic steatosis is also associated with significant liver disease in some patients. As many as 10-20% of patients with NAFLD develop steatohepatitis [1] and approximately 5% proceed to liver cirrhosis within 10 years of diagnosis [2].

The hallmark of hepatic steatosis is the presence of triglycerides (TGs) stored as large lipid droplets in the cytoplasm of hepatocytes. Hepatic steatosis strongly associates with hypertriglyceridaemia and low high density lipoprotein cholesterol (HDL-C) levels, two common features of the metabolic syndrome. The vast majority of individuals with hepatic steatosis have elevated alanine transaminase (ALT) and consequently ALT has been used as a surrogate index of NAFLD [3]. Hypertriglyceridaemia is also strongly associated with elevated transaminases, in particular ALT [4].

Recently, it has been proposed that two sequence variants in the promoter of the gene encoding human apolipoprotein C3 (*APOC3*, rs2854116 and rs2854117) were associated with hypertriglyceridaemia, nonalcoholic fatty liver disease and insulin resistance in lean individuals of South Asian descent [5]. Apolipoprotein C3 is a small exchangeable apolipoprotein (79 amino acids) and one of the major constituents of plasma very low density lipoprotein (VLDL), chylomicrons and HDL-C [6]. Elevated plasma apolipoprotein C3 levels are positively correlated with plasma triacylglycerol concentrations in hypertriglyceridemic subjects [7]. Apolipoprotein C3 inhibits the hydrolysis of TG-rich particles by lipoprotein lipase and their apoE-mediated hepatic uptake [8,9]. Experimental studies have demonstrated that overexpression of APOC3 causes delayed clearance of TG-rich lipoproteins from plasma, resulting in hypertriglyceridaemia [6]. The suggested mechanism involves therefore increased plasma concentrations of apolipoprotein C3, which in turn inhibits lipoprotein lipase and triglyceride clearance, and consequently increases fasting and post-prandial hypertriglyceridaemia.

More recent data do not confirm the association between the rs2854116 and rs2854117 variants in APOC3 and either hepatic triglyceride content or insulin resistance or fasting triglyceride levels in a large population-based (n = 2,239) study comprising three different ethnic groups (European Americans, African Americans, and Hispanics) [10]. In particular, authors failed to replicate the association between the APOC3 SNPs and hepatic triglyceride content measured by magnetic resonance spectroscopy [10].

We have previously observed that excess of body mass exacerbates the association of the Patatin like phospholipase domain containing C 3 protein (PNPLA3) I148M variant with hepatic fat and liver damage, with increased circulating ALT levels, in a cohort of severly obese Italian adults and children [11,12]. The role of BMI in increasing ALT levels and the risk of NAFLD has been recognized for several years [13]. In this contex we hypothize that obesity would expose the association between the APOC3 variants and liver parameters, insulin-resistance and/or lipid levels.

We hence genotyped a large cohort (n = 585) of Italian obese subjects (mean body mass index (BMI) = 42 ± 8 kg/m^2^) for the two sequence variants in APOC3 gene promoter (rs2854116 [-455 T/C] and rs2854117 [-482 C/T]), aiming to evaluate the association between APOC3 variants and alanine transaminase levels, triglyceride levels and insulin resistance, in subjects with a higher predisposition to an altered metabolic profile.

## Methods

### Patient recruitment

A total of 585 consecutive unrelated obese (BMI ≥ 30 kg/m^2^) Caucasians were recruited from the Day-Hospital of the Department of Clinical Sciences, University of Rome "La Sapienza". They underwent complete medical evaluation and a standard 75 g oral glucose tolerance test (OGTT) with measurements of glucose and insulin at baseline and after 30, 60, 90, 120 minutes. Individuals with daily ethanol consumption of greater than 40 grams or a history of viral hepatitis were excluded from the study. Metabolic Syndrome was classified according to NCEP ATP-III 2004 criteria. In the recruitment of our cohort alcohol consumption was classified as a categorical variable (> or < 40gr) based on the International Center for Alcohol Policies (ICAP, http://www.icap.org/). This organization reports the recommended alcohol intake for Italy (as stated by the Italian Ministry for Agriculture & Forestry and National Institute for Food and Nutrition) to be < 40 g/day with no differences between men and women (http://www.icap.org/Table/InternationalDrinkingGuidelines).

In a subgroup (n = 170) of our study population ultrasound imaging for liver fat content was available. Ultrasound scanning was performed by the same operator, who was unaware of the aims of the study and blinded to laboratory values. Liver steatosis was assessed as present or absent. Steatosis was determined on the basis of abnormally intense, high-level echoes arising from the hepatic parenchyma, liver-kidney differences in echo amplitude, echo penetration into deep portions of the liver and clarity of vascular structures.

Participants were not on glucose lowering drugs. Also patients in lipid-lowering treatment were excluded from the study. All individuals provided informed consent prior to inclusion in the study. Research carried out on humans complied with the Helsinki Declaration. The study was approved by the University research ethics committee.

#### Biochemistry

Glucose, insulin, cholesterol, HDL-choleserol and triglycerides (TGs) were measured as previously described [14], after an overnight fast. Glucose leveles below 100 mg/dl, HDL > 40 mg/dl for men and > 50 mg/dl for women, triglycerides < 150 mg/dl were taken as normal limits.

Serum alanine (ALT) and aspartate aminotransferase (AST) levels in fasting subjects were assayed using a Hitachi 737 analyzer (Boehringer-Mannheim Diagnostics, Indianapolis, IN). HOMA-IR and Insulin Sensitivity Index (ISI) indices were calculated as previously shown by Matthews et al. and Matsuda et al. [15,16].

#### Genotyping assay

We developed fluorogenic 5'-nucleotidase assays for APOC3 rs2854117 gene polymorphism. The assay was performed using the TaqMan C7241_10 assay (Applied Biosystems, Foster City, CA) in a total volume of 2.7 μl on a 7900HT Fast Real-Time PCR instrument (Applied Biosystems). The plate was run at 95°C for 10 minutes, 92°C for 15 seconds then 60°C for 1 minute for 40 cycles.

The *APOC3 *rs2854116 gene variant was assayed by the restriction fragment length polymorphism (RFLP) technique. A 497-bp fragment was amplified and enzymatically restricted using *BtsCI *(New England Biolabs, Ipswich, MA, USA). The resulted enzyme-digested fragments of the PCR products were fractionated on 3.5% agarose gels, stained with ethidium bromide, and visualized with an imaging system (Amersham-Pharmacia Biosciences).

Allele frequencies were in Hardy-Weinberg Equilibrium.

### Statistical analysis

Categorical variable distributions were compared by the Pearson χ2. Differences between continuous variables across the genotype classes were evaluated by ANOVA including gender, age and BMI as covariates. Skewed variables wer logharitmically transformed prior to entering the analyses. The study had a power of 80% at an error rate of 5% to allow detection of differences in triglycerides or ALT/AST levels (the main variables associated with APOC3 gene [5]). Linkage disequilibrium between the rs2854116 and rs2854117 SNPs was assessed by calculating the disequilibrium statistics Δ [17] and D' [18]. The sign of D' (positive or negative) depends on the arbitrary choice of the alleles paired at the two loci, and indicates whether the same or opposite allelic association is present. All statistical analyses were performed with SPSS 17.0 statistical package.

## Results

Clinical characteristics of the study subjects are reported in Table 1. All subjects were obese (median BMI 41 kg/m^2^, interquartile range 36.5-47.5) Mean blood pressure, median lipid and glucose parameters were within normal ranges. Only insulin resistance indices (HOMA-IR and ISI) were indicative of reduced insulin sensitivity, as would be expected in obese subjects. Looking at subjects with an altered lipid profile, 27% had high TGs. Low HDL was present in 32.7% of males and 50.6% of females, and 16% had both, high TGs and low HDLs, an association also defined as the atherogenic dyslipidaemia. Median transaminase levels were also within normal limits (using < 40 U/L as cut-off) (Table 1).

**Table 1:** Clinical characteristics of study subjects

N	585
**Women/Men**	422/163
**Age (yr)**	42 (31-52)
**Body Mass Index (Kg/m^2^)^a^**	41 (36.5-47.5)
**SBP (mm/Hg)**	130 (120-140)
**DBP (mm/Hg)**	80 (80-90)
**Triglycerides (mg/dL)**	113 (81-157.2)
**Total Cholesterol (mg/dL)**	200 (178-227.1)
**HDL Cholesterol (mg/dL)**	Male: 43 (38.1-49.1) Female: 50 (44-58.5)
**LDL Cholesterol (mg/dL)**	122 (103.5-146.2)
**ALT (U/L)**	28 (20.5-43)
**AST (U/L)**	20 (16-26)
**Fasting blood glucose (mg/dL)**	90 (82-99)
**Fasting blood insulin (μU/mL)**	24 (16.3-35)
**HOMA-IR (U)**	5.1 (3.4-8.4)
**ISI (U)**	2.1 (1.4-3.4)
**Type 2 diabetes n (%)**	40 (6.8)

^a ^BMI was calculated as body weight (kg)/height (m^2^). Values are medians and interquartile ranges. Abbreviations: SBP, systolic blood pressure; DBP, diastolic blood pressure; HDL, high density lipoprotein; LDL, low density lipoprotein; ALT, alanine transaminase; AST, aspartate transaminase; HOMA-IR, homeostatic model assessment of insulin resistance; ISI, insulin sensitivity index.

The allele frequencies of the rs2854117 (-482T) and rs2854116 (-455C) APOC3 alleles were 0.26 and 0.37, respectively, similar to previously reported frequencies [10]. Linkage disequilibrium between the two SNPs by the disequilibrium statistics Δ and D' showed a very strong linkage disequilibrium (D' = -0.97, Δ = 0.43, P < 0.0001).

As previously analyzed [5] we first divided the study subjects into two groups: those who were homozygous for the wild-type alleles at both SNPs (-482C and -455T alleles) and those who were carriers of at least one allele or both (-482T, -455C or both). The rationale for this is the association between the variant alleles at rs2854116 (T-455C) and at rs2854117 (C-482T) have been shown to be independently associated with higher APOC3 levels [19]. We next analyzed the two variants separately in our study cohort.

The clinical parameters of the study subjects, stratified by genotype classes are shown in Table 2. There were no significant differences between the two groups in plasma concentration of ALT and aspartate transaminase (AST) levels, total cholesterol, HDL-and low density lipoprotein (LDL)-cholesterol, and circulating triglycerides (Table 2).

**Table 2:** Clinical Features of the Study Participants in APOC3 Wild-Type Homozygotes (C-482 and T-455) and Carriers of Variant Alleles (C-482T, T-455C or both)

	Wild Type Homozygotes (n = 239)	Variant-Allele Carriers (n = 346)	p value^a^	
			Linear regression	Mann-Whitney Test
**ALT (U/L)**	27 (20-41.5)	29 (21-44)	0.102	0.125
**AST (U/L)**	19 (16-25)	20 (16-26.7)	0.190	0.366
**Triglycerides (mg/dL)**	115 (79.9-158.1)	112 (81.6-157)	0.575	0.919
**Total Cholesterol (mg/dL)**	200 (178.3-229)	200 (178-224.4)	0.726	0.698
**HDL Cholesterol (mg/dL)**	48 (42.4-56)	48 (41-56.5)	0.617	0.509
**LDL Cholesterol (mg/dL)**	122 (105.2-149.5)	125 (100.3-145.3)	0.758	0.711
**Age (yr)**	42 (33-53)	42 (30-51)	0.200	0.261
**Body Mass Index (Kg/m^2^)**	41 (37-47.3)	41 (36-47.7)	0.813	0.575
**Fasting blood glucose (mg/dL)**	90 (83-98)	89 (81-100)	0.985	0.697
**Fasting blood insulin (μU/mL)**	23 (15.4-33)	25 (16.6-36.6)	0.371	0.294
**HOMA-IR (U)**	5 (3.4-7.7)	5.1 (3.4-8.8)	0.341	0.361
**ISI (U)**	2.1 (1.4-3.6)	2.1 (1.3-3.3)	0.925	0.609

^a ^P values were calculated using linear regression model including age, BMI and sex. Fasting blood glucose, fasting blood insulin, HOMA-IR and ISI were log-transformed before being entered into the model. Values are medians and interquartile ranges.

**Figure 1:**
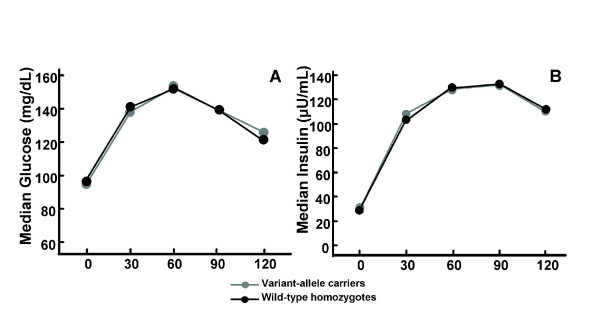
**Carriers of APOC3 variant alleles (-482T, -455C or both) were compared with homozygous for the wild-type alleles at both SNPs (-482C and -455T alleles) for glucose and insulin measurements during oral glucose tolerance test (OGTT)**. The study cohort underwent OGTT (75 g) test and glucose and insulin were measured baseline (0) and at 30, 60, 90 and 120 min after the administration of glucose. (A) Median glucose and (B) insulin in individuals stratified by the genotypes. No statistical differences were found in either insulin or glucose at different time points in the two groups. P-values were calculated by linear regression (all P-values = NS), including age, gender and body mass index (BMI) as covariates in the model.

We also tested for an association with surrogate indices of insulin resistance (homeostatic model assessment of insulin resistance and Matsuda Insulin Sensitivity Index), but no differences were found in the 340 variant-allele carriers compared to the wild-type homozygote subjects (Table 2). Glucose and insulin levels were similar in the two groups throughout the oral glucose tolerance test (Figure 1).

To confirm the lack of effects of the two SNPs, we evaluated the prevalence of variant alleles in subjects with normal and pathological serum levels of ALT (defined as > 40 U/L). In individuals with elevated levels of ALT, we found no significant difference in the variant allele frequency 40.6% vs 59.4% (wild type homozygotes vs variant-allele carriers respectively, P = 0.918) (Table 3).

**Table 3:** Comparison of prevalence of variant alleles estimated by chi-square analyses in subgroups

	Wild Type Homozygotes n (%)	Variant-Allele Carriers n (%)	p value^a^
**ALT ≥ 40 (U/L)**	54 (40.6)	79 (59.4)	0.918
**ALT < 40 (U/L)**	145 (41.4)	205 (58.6)	

**75^th ^HOMA-IR (U)**	48 (36.6)	83 (63.4)	0.512
**25^th ^HOMA-IR (U)**	55 (41.4)	78 (58.6)	

**Liver steatosis**	49 (42.6)	66 (57.4)	0.869
**No liver steatosis**	25 (45.5)	30 (54.5)	

^a ^P values were calculated using the Pearson χ2. Abbreviations: ALT, alanine transaminase; HOMA-IR, homeostatic model assessment of insulin resistance.

Furthermore, we investigated the prevalence of variant alleles by comparing the low and the high ends of HOMA-IR index distribution. No significant difference in the variant allele frequency 41.4% vs 58.6% (wild type homozygotes vs variant-allele carries respectively) in the lowest quartile of HOMA-IR, and 36.6% vs. 63.4% (wild type homozygotes vs variant-allele carries respectively) in the highest quartile of HOMA-IR (P = 0.512) (Table 3).

We then examined the three genotypes in each SNP independently. Neither the rs2854117 nor the rs2854116 APOC3 polymorphism associated with any of the parameters tested (data not shown) confirming the lack of effect of the variants. Further anlayses with the haplotypes groups derived from rs2854117 and rs2854116 APOC3 polymorphisms confirmed the lack of association of the APOC3 variants with clinical and metabolic parameters (data not shown).

To exclude that the presence of insulin-resistance or obesity could mask the possible effects of the APOC3 SNPs, we stratified our subjects on the basis of their insulin-resistance indices, insulin levels, or grade of obesity. When we selected the carriers (n = 78) of the -482T, -455C or both SNPs within the most insulin-sensitive (lowest quartile of HOMA-IR), we did not observe any effect on transaminases and on lipoprotein levels (data not shown). Similar negative results were observed when subjects were selected from those with the highest (34.8 μU/mL) levels of plasma insulin. Among our cohort of 585 obese individuals 53% (n = 311, BMI > 40 kg/m^2^) were morbidly obese and 47% were only obese (n = 274) with BMI ranging between 30 and 40 kg/m^2^. When we performed the same genetic analyses in the two separate groups, we did not find any difference in mean levels of ALT and/or AST, triglycerides and insulin resistance parameters. No differences were found when the obese subjects were stratified by gender (data not shown). Also, no differences in any parameter were observed between carriers of the APOC3 variants and non-carriers when we analysed in our cohort only the subjects with the Metabolic Syndrome (n. = 261) (data not shown).

Finally, To account for other possible confounding factors such as use of non-steroidal anti-inflammatory (NSAIDs) and antihypertensive drugs, we looked at transaminase levels in individuals treated with these drugs and untreated patients. No differences in ALT/AST levels were observed for both classes of drugs, nor the genetic study was modified when a separate analyses excluding those under NSAIDs/antipypertensive treatment was performed (data not shown).

A subgroup (n = 170) of our study population was analyzed for liver fat content by ultrasound scanning. We observed a frequency of 68% of steatosis. When, we compared ALT levels between individuals with no liver damage vs patients with diagnosis of steatosis we observed a significant increase in transaminase levels (27.3 ± 14.5 vs 37 ± 26.7 U/L respectively, P = 0.009) with a significant correlation (p < 0.005), confirming alanine transaminase as a reliable marker of liver injury in our population. When we performed separate analyses in this subset of individuals, we found very similar results as observed in the whole studied population. There were no significant differences between the two genotype classes in plasma concentration of ALT and AST levels, presence or absence of steatosis, total cholesterol, HDL-and low density lipoprotein (LDL)-cholesterol, circulating triglycerides, surrogate indices of insulin resistance (homeostatic model assessment of insulin resistance and Matsuda Insulin Sensitivity Index), glucose and insulin levels (data not shown). Furthermore, we found no significant difference in the variant allele frequency (42.6% wild type homozygotes vs 57.4% variant-allele carries respectively, P = 0.869) in the subjects with echographic diagnosis of liver steatosis (Table 3).

## Discussion

Genetic susceptibility may be influenced by obesity status. Previous studies have described modulation of the PPARG Pro12Ala SNP by BMI in type 2 diabetes [20,21]. Also, we have recently observed that excess of body mass exacerbates the association of the PNPLA3 I148M variant with hepatic fat and liver damage, with increased circulating ALT levels, in a cohort of Italian severe obese subjects (mean body mass index (BMI) = 41 kg/m^2^) [11]. The association between the PNPLA3 variant and liver damage was also confirmed in obese children [12].

Similarly, in this study we performed genetic association analyses in a cohort of Italian obese subjects, to test whether obesity would expose the association between the APOC3 rs2854116 and rs2854117 variants and ALT levels as surrogate markers of hepatic steatosis. Also triglyceride levels and indices of insulin-resistance were analyzed for association.

We did not observe any significant difference between carriers of the variants and wild-type homozygote subjects in plasma concentrations of ALT and/or AST, thus suggesting that the two SNPs are not influencing fatty liver disease in our obese cohort. Although transaminases are only surrogate indices of fatty liver disease, in many epidemiological studies ALT, in particular, has been used as a marker of liver fat accumulation [4,22,23] and is commonly used in clinical practice as marker of steatohepatitis [24]. Finally, ALT levels strongly correlate with BMI, but increased ALT in overweight persons should not be interpreted as nonspecific biochemical interference and corresponds to typical histopathologic lesions [25].

In addition, we did not find any association between the APOC3 SNPs and circulating triglycerides, total cholesterol, HDL-and LDL-cholesterol. Petersen et al. [5] found that fasting plasma triglyceride levels were approximately 60% higher in the carriers of the variant alleles in lean Asian Indian men. However, Richart and colleagues recently found no relationship between APOC3 mRNA expression and triglycerides content in the livers of 44 morbidly obese women of European descent. Also they did not find any association between gene expression and plasma triglyceride concentrations or insulin-resistance index determined by homeostasis model assessment [26]. Very recent data from a large multiethnic population in which hepatic triglyceride content was measured by magnetic sprectoscopy did not replicate the association between these two variants in APOC3 and either hepatic triglycerides content or insulin resistance [10]. These results are consistent with the lack of association in transaminase levels in obese carriers that we observe in this study.

Expression studies showed that APOC3 gene is transcriptionally downregulated by insulin [27]. Furthermore it was shown that, unlike the wild-type promoter, the promoter containing variants at positions -455 and -482 remains constitutively active over a high range of insulin concentrations with a reduced affinity for the nuclear transcription factors mediating the insulin response, thus inducing insulin resistance at the gene level [19]. It could be argued that, in the context of the insulin resistance associated with obesity the APOC3 variants are unable to induce further actions on lipid levels. However, we should point out that when we looked only at carriers of the variant alleles with normal insulin sensitivity, we did not observe any association with indices of fatty liver disease and lipid levels, suggesting that also in this state the APOC3 variants are not influencing TG concentrations.

One limitation of our study is that we do not have direct measurement of hepatic fat content (echography or MRI) in all our study subjects. However, as discussed above, ALT is a well established variable to asses liver damage [4,22,23] and it is commonly used in clinical practice as a marker of steatohepatitis [24,28,29]. Furthermore, when we compared in a subset of subjects ALT levels between individuals with no liver damage vs patients with diagnosis of steatosis by echography, we observed a significant increase in transaminase levels confirming alanine transaminase as a reliable marker of liver injury in our population. The genetic analyses in this subgroup confirmed the lack of association of the APC3 variants. Another possible limitation relates to the alcohol consumption limits proposed by WHO, which is different according to gender. However, in the recruitment of our cohort the alcohol consumption was classified as > or < 40 gr based on the International Center for Alcohol Policies (ICAP), which does not establish any difference between men and women in the Italian population.

Another point to bear in mind is that all studies on APOC3 have been performed in different ethnic groups and in different countries with large differences in dietary habits. However, the lack of association of APOC3 variants has been now consistently reported in European American, African American, and Hispanic populations.

## Conclusions

In summary, no significant association between the rs2854116 and rs2854117 genetic variants in APOC3 gene and impairment in markers of fatty liver disease, lipid profile and insulin-resistance was observed in our Italian obese population, thus not confirming the suggested role [5] of these APOC3 gene sequence variants.

## List of abbreviations

NAFLD: non-alcoholic fatty liver disease; ALT: alanine transaminase; AST: aspartate transaminase; BMI: body mass index; HOMA-IR: homeostasis model assessment for insulin resistance; ISI: Insulin Sensitivity Index; TG: triglycerides; APOC3: apolipoprotein C3; OGTT: oral glucose tolerance test.

## Competing interests

The authors declare that they have no competing interests.

## Authors' contributions

The study was designed by FS, SR and MGB. FS and SR equally contribuited to the work. All subjects data were obtained by CM, MI, MAB, EC and FL. The database organization was carried out by FS and FC. Experimental data was obtained by FS and SR. Data analyses were performed by FS and MGB. The paper was written by FS and MGB and all authors read and approved the final manuscript.
